# High-density lipoprotein cholesterol and cognitive impairment: A U-shaped relationship in China’s aging population

**DOI:** 10.1371/journal.pone.0343768

**Published:** 2026-03-25

**Authors:** Fei Wang, Xiang Shang, Qiqi Yang, Zhuang Tao, Weimin Li, Meixia Wang, Fei Li

**Affiliations:** 1 The First Clinical Medical College of Anhui University of Chinese Medicine, Hefei, China; 2 The First Affiliated Hospital of Anhui University of Chinese Medicine, Hefei, China; 3 Medical school, Hefei Technology College, Hefei, China; 4 Second Affiliated Hospital of Anhui University of Chinese Medicine, Hefei, China; Sarich Neuroscience Research Institute, AUSTRALIA

## Abstract

This study investigated the association between high-density lipoprotein cholesterol (HDL-C) levels and the risk of cognitive disorders in older adults. Data were obtained from the 2011 Chinese Health and Retirement Longitudinal Study and included 7,509 participants. Cognitive function was assessed using a scale that measured episodic memory and mental status. Statistical analyses included multiple linear regression, restricted cubic splines, and threshold effect analysis to explore the relationship between HDL-C levels and cognitive scores. Compared with Q1 (<35 mg/dL), very high HDL-C was associated with lower cognitive scores (Q4: β = −0.622 [95% CI, −0.908 to −0.337]; Q5: β = −0.322 [−0.627 to −0.017]). A U-shaped association was observed, with a turning point at 67.43 mg/dL. Below the threshold, a 1-SD higher HDL-C was associated with a 0.08-SD higher cognitive score (β = +0.08; 95% CI, 0.06–0.11; p < 0.001), whereas above the threshold a 1-SD higher HDL-C was associated with a 0.07-SD lower score (β = −0.07; 95% CI, −0.14 to −0.01; p = 0.019). Therefore, the relationship between lipid profiles and cognitive health is nuanced and nonlinear. Understanding these complexities is crucial for developing strategies to maintain cognitive function in older adults.

## 1. Introduction

Cognitive impairment is categorized according to its severity as either moderate cognitive impairment or dementia. Mild cognitive impairment is an intermediary condition in which cognitive performance lies between that of normal function and dementia [1]. Recent estimates indicate that the global population of individuals living with dementia is expected to increase significantly, from approximately 55 million in 2019–139 million by 2050 [2]. A nationwide cross-sectional survey in China indicated that cognitive impairment impacts 22.24% of individuals aged 60 and above, which is the highest globally [3,4]. Currently, there is no effective targeted treatment for the intricate origins and latent early manifestations of dementia [5]. Consequently, early identification of risk factors and proactive management are of substantial therapeutic importance.

HDL cholesterol can impede the development of atherosclerosis through multiple mechanisms and is regarded as a preventive factor against cardiovascular and cerebrovascular disorders [6,7]. Recent studies indicate that elevated HDL levels may paradoxically elevate the incidence of cardiovascular disease and augment the risk of all-cause death [8–10]. Cohort studies have indicated that elevated HDL levels may also increase the incidence of some non-cardiovascular diseases, including specific digestive system cancers (such as gastroesophageal and liver cancers), diabetes, and particular musculoskeletal disorders [11–14]. This may be linked to the association of excessively elevated HDL-C levels with cholesterol overload and malfunction, which subsequently promotes inflammation [15–17]. Considering that chronic inflammation is a significant contributor to numerous age-related disorders, including dementia [18], it may be hypothesized that excessively elevated HDL-C levels may negatively affect cognitive performance in older adults.

Preliminary studies indicate that increased blood HDL-C levels may enhance cognitive performance and decrease the risk of cognitive decline, whereas low HDL-C levels are linked to a greater incidence of cognitive impairments in paediatric cancer survivors [19–21]. A study in the United States revealed that both abnormally high and low HDL-C levels may increase the risk of cognitive impairment [22]. Moreover, certain studies have identified no substantial correlation between HDL-C levels and cognitive impairments following traumatic brain damage [23]. Nonetheless, observational studies have not yet yielded conclusive data concerning the association between HDL-C levels and cognitive impairment. Therefore, it is crucial to understand how HDL-C affects cardiovascular and cerebrovascular diseases in older adults. Additional investigation into the possible association between elevated HDL-C levels and the risk of cognitive impairment in middle-aged and older individuals could improve our understanding of this connection.

Discourse regarding the relationship between lipids and cognitive function persists, and a consensus is yet to be reached in this domain. This study employed longitudinal datasets from the China Health and Retirement Longitudinal Study (CHARLS) to investigate the relationship between HDL-C levels and cognitive impairment in middle-aged and elderly Chinese individuals.

## 2. Materials and methods

### 2.1. Study design and participants

The data for this cohort study were obtained from the CHARLS. This was an extensive longitudinal study undertaken by Peking University in partnership with Wuhan University and other institutions. CHARLS is a comprehensive, interdisciplinary survey that offers high-quality, nationally representative data regarding the familial and personal situations of Chinese adults aged ≥ 45 years. The research commenced with a preliminary survey in 2008 and succeeded in collecting national baseline data in 2011. The project was implemented in 150 counties and districts, encompassing 450 rural and urban regions, and involving 10,257 homes and 17,708 participants. Follow-up surveys were conducted biennially and triennially [24]. The Peking University Biomedical Ethics Committee approved the CHARLS program (IRB0000001052–11015), and all individuals taking part in the research gave their written informed consent.

**Fig 1:**
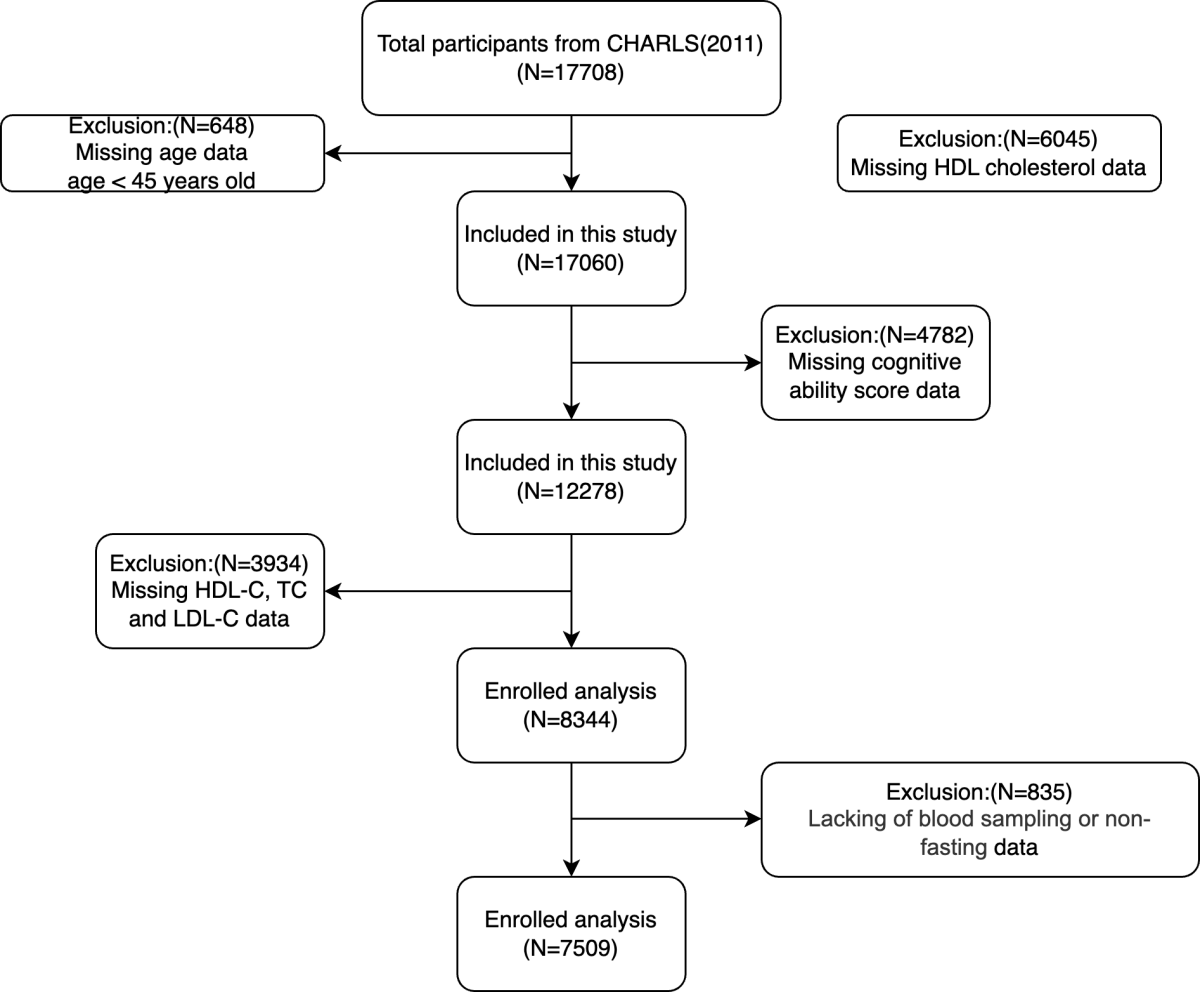
Participant selection flowchart.

This study used data from the CHARLS 2011 baseline survey, which encompassed cognitive evaluation and blood collection. Eligibility required participants to be 45 years or older and possess accessible HDL-C data. The exclusion criteria were age < 45 years; absence of age data; absence of HDL-C, total cholesterol, or triglyceride data; absent cognitive function score; and non-fasting status during blood collection without accessible fasting data. This study involved 7,509 participants to investigate the correlation between HDL-C levels and cognitive impairment. Fig. 1 illustrates the participant selection process.

### 2.2. Measurement of baseline serological indicators

Physicians involved in venous blood collection received standardized training by the China Centers for Disease Control and Prevention (China CDC). Fasting venous blood was drawn into K2EDTA (EDTA-K2) anticoagulant vacuum tubes and promptly processed to separate plasma. Plasma aliquots were transported on dry ice to the China CDC biobank and stored at −80°C until assay. Subsequent biochemical analysis (including the HDL-C assay) were conducted at the You’anmen Clinical Laboratory Center of Capital Medical University under routine internal and external quality-control procedures [25]. HDL-C was measured using a direct enzymatic method under standardized quality-control procedures. Approximately 92% of participants self-reported fasting at the time of blood draw.

### 2.3. Cognitive function assessment

Based on a previous CHARLS-based study [26], we used the Cognitive Abilities Assessment Scale, which comprises situational memory and mental-state components, to evaluate cognitive function. Memory performance in situational circumstances was evaluated by tests requiring immediate and delayed word recall, in which participants were instructed to memorise and reproduce a list of 10 specified terms. The scores for these two tasks were averaged to obtain a total score between 0 and 10. The mental-state component included the TICS-10 and drawing tasks. The TICS-10 evaluates the participant’s capacity to subtract 7 from 100 (up to five iterations), he evaluation encompassed ascertaining the present date (month, day, and year), in addition to identifying the day of the week and the season. In the figure-drawing exercise, participants were instructed to replicate two crossing pentagons, with scores spanning from 0 to 11. The overall cognitive score ranged from 0 to 21, with elevated levels indicating superior cognitive function [27].

### 2.4. Participant characteristics

We incorporated demographic features and health-related factors from the 2011 baseline survey into the study, as indicated in previous studies. Demographic factors included sex (male or female), age, educational attainment (illiterate, primary, secondary, and higher), and marital status (married or other, including divorced, widowed, or never married). Health-related factors included body mass index (BMI), hypertension, diabetes, depression, smoking, smoking status, and drinking status.

### 2.5. Statistical analysis

Linear regression analysis was utilised to evaluate the relationship between baseline HDL-C levels and cognitive score. The selection of covariates for adjustment in the multivariate models was based on background information, existing literature, and data availability [28]. In the regression analyses, adjustments were made for variables associated with exposure, outcomes, or both [29]. In Model II, we controlled for age (categorized as 45–60 years and ≥60 years) and sex. Model III was adjusted for educational level, marital status, hypertension, diabetes, depression, current alcohol consumption, current smoking, socialization, total cholesterol, and triglyceride levels. The restricted cubic spline (RCS) method was employed to investigate potential nonlinear relationships between HDL levels and the risk of cognitive impairment, using 50 mg/dL (the median value in the research group) as the reference point in Model III. Threshold effect assessments were conducted to identify possible turning points. Subgroup evaluations utilised covariates modified in Model III, employing standardised continuous predictors, to examine their influence on the relationship between HDL-C levels and the likelihood of cognitive deterioration. To consider HDL-C within the broader lipid profile (including LDL-C), we performed a sensitivity analysis using the non-HDL-C/HDL-C ratio (NHHR), calculated as (total cholesterol − HDL-C)/HDL-C. NHHR models were adjusted for the same covariates as Model III, except total cholesterol was not additionally adjusted because it is part of the NHHR construct.

## 3. Results

### 3.1. Baseline characteristics of participants categorized by HDL-C percentiles

The 10th and 90th HDL-C level percentiles were 33.25 mg/dL and 70.36 mg/dL, respectively. Participants were categorized into five groups based on references 29 and 30: Q1 (<35 mg/dL), Q2 (35–40 mg/dL), Q3 (40–60 mg/dL), Q4 (60–70 mg/dL), and Q5 (≥70 mg/dL). Table 1 shows the baseline patient characteristics.

**Table 1:** Analysis of HDL-C values based on demographic and clinical factors.

Variables	HDL-C categories (mg/dL)					
	Q1 (<35)	Q2(35–40)	Q3(40–60)	Q4(60–70)	Q5 (≥70)	*p*-value
N	989	883	3860	983	794	
Age (years)	58.59 (8.75)	58.39 (8.74)	58.54 (8.92)	59.43 (9.37)	59.85 (9.17)	<0.001***
Age group, n (%)						0.009*
45–60	574 (58.04)	510 (57.76)	2228 (57.72)	537 (54.63)	408 (51.39)	
≥60	415 (41.96)	373 (42.24)	1632 (42.28)	446 (45.37)	386 (48.61)	
Sex, n (%)						<0.001***
Male	409 (41.35)	406 (45.98)	2023 (52.41)	529 (53.81)	382 (48.11)	
Female	580 (58.65)	477 (54.02)	1837 (47.59)	454 (46.19)	412 (51.89)	
Education, n (%)						<0.001***
Illiteracy	349 (35.29)	327 (37.03)	1575 (40.80)	455 (46.29)	374 (47.10)	
Primary school	231 (23.36)	204 (23.10)	915 (23.70)	223 (22.69)	187 (23.55)	
Middle school and above	409 (41.35)	352 (39.86)	1370 (35.49)	305 (31.03)	233 (29.35)	
Marital status, n (%)						0.026
Married	91 (9.20)	95 (10.76)	388 (10.05)	118 (12.00)	105 (13.22)	
Others	898 (90.80)	788 (89.24)	3472 (89.95)	865 (88.00)	689 (86.78)	
Hypertension, n (%)						<0.001***
No	483 (48.84)	443 (50.17)	2092 (54.20)	576 (58.60)	462 (58.19)	
Yes	506 (51.16)	440 (49.83)	1768 (45.80)	407 (41.40)	332 (41.81)	
Diabetes, n (%)						<0.001***
No	716 (72.40)	708 (80.18)	3291 (85.26)	885 (90.03)	715 (90.05)	
Yes	273 (27.60)	175 (19.82)	569 (14.74)	98 (9.97)	79 (9.95)	
Depression, n (%)						<0.001***
No	668 (67.54)	616 (69.76)	2485 (64.38)	610 (62.05)	481 (60.58)	
Yes	321 (32.46)	267 (30.24)	1375 (35.62)	373 (37.95)	313 (39.42)	
Socializing, n (%)						0.003**
No	441 (44.59)	385 (43.60)	1858 (48.13)	479 (48.73)	412 (51.89)	
Yes	548 (55.41)	498 (56.40)	2002 (51.87)	504 (51.27)	382 (48.11)	
Current smoking, n (%)						0.017**
No	661 (66.84)	601 (68.06)	2677 (69.35)	689 (70.09)	506 (63.73)	
Yes	328 (33.16)	282 (31.94)	1183 (30.65)	294 (29.91)	288 (36.27)	
Current drinking, n (%)						<0.001***
No	664 (67.14)	588 (66.59)	2602 (67.41)	596 (60.63)	419 (52.77)	
Yes	325 (32.86)	295 (33.41)	1258 (32.59)	387 (39.37)	375 (47.23)	
TC (mg/dL)	182.58 (39.68)	188.69 (38.51)	193.53 (37.19)	199.96 (35.36)	208.20 (34.77)	<0.001***
TG (mg/dL)	236.32 (161.61)	163.19 (88.64)	119.12 (62.43)	89.69 (41.88)	78.19 (35.44)	<0.001***
HDL-C (mg/dL)	29.79(4.56)	37.63(1.43)	49.44(5.59)	64.52(2.91)	81.00(10.48)	<0.001***
LDL-C (mg/dL)	99.13 (37.18)	116.41 (33.54)	121.98 (33.65)	120.66 (33.06)	114.93 (33.00)	<0.001***
BMI (kg/m^2^)	25.24 (3.64)	24.86 (3.68)	23.70 (3.68)	22.40 (3.29)	21.65 (3.17)	<0.001***
Cognitive score	12.48(3.36)	12.35(3.56)	11.98(3.51)	11.35(3.65)	11.56(3.65)	<0.001***

Continuous data were presented as means (SD), and categorical data were presented as numbers (%).

* *p* < 0.05, ** *p* < 0.01, *** *p* < 0.001.

Compared to the other groups, the Q5 group displayed a higher proportion of female participants, persons lacking formal education, and those with non-traditional marital statuses. In contrast, this group had a reduced percentage of people with hypertension, diabetes, depressive symptoms, engagement in social activities, smoking behaviours, and alcohol intake. The average age of the Q5 group was higher than that of the other groups. The proportion of illiterate individuals with primary education in Q5 exceeded that of the other groups, whereas the proportion of individuals with secondary education and higher in Q5 was lower than that of the other groups. Participants in groups Q4 and Q5 exhibited lower cognitive function scores than those in group Q1.

### 3.2. Multivariate regression analysis of HDL-C and cognitive impairment

Table 2 illustrates the correlation between HDL-C levels and the risk of cognitive deterioration. In multivariable-adjusted models (Model III), compared with the reference group Q1 (<35 mg/dL), the intermediate categories Q2 (35–40 mg/dL) and Q3 (40–60 mg/dL) did not differ significantly in cognitive score. In contrast, very high HDL-C categories showed lower cognitive scores relative to Q1: Q4 (60–70 mg/dL), β = −0.622 (95% CI, −0.908 to −0.337; p < 0.001) and Q5 (≥70 mg/dL), β = −0.322 (95% CI, −0.627 to −0.017; p = 0.039). These β values reflect point differences (score units) versus Q1, not percentages. Because Q1 is the lowest HDL-C group and serves as the reference, category contrasts do not test risk at low HDL-C; therefore, the lack of difference for Q2–Q3 does not contradict the U-shaped pattern observed in continuous models.

**Table 2:** Correlation between initial HDL-C levels and the probability of cognitive deterioration throughout subsequent follow-up intervals^a^.

Exposure	Model I	Model II	Model III
	β (95% CI), *p*-value	β (95% CI), *p*-value	β (95% CI), *p*-value
HDL-C quartiles			
Q1	0.00 (Reference)	0.00 (Reference)	0.00 (Reference)
Q2	−0.122 (−0.441–0.198) 0.455	−0.061 (−0.369–0.248) 0.699	−0.085 (−0.356–0.186) 0.539
Q3	−0.495 (−0.741 – −0.49) <0.001***	−0.355 (−0.593 – −0.117) 0.004**	**−0.222 (−0.445–0.001) 0.052**
Q4	−1.122 (−1.433 – −0.812) <0.001***	−0.918 (−1.219 – −0.618) <0.001***	−0.622 (−0.908 – −0.337) <0.001***
Q5	−0.911 (−1.240 – −0.583) <0.001***	−0.729 (−1.047 – −0.411) <0.001***	−0.322 (−0.627 – −0.017) 0.039*
*p* for trend	<0.001***	<0.001***	0.004**

Model I: Crude.

Model II: Adjusted for age and sex.

Model III: Adjusted for age, sex, education, marital status, hypertension, diabetes, depression, drinking status, smoking status, socialization, total cholesterol level, and triglyceride level.

CI: Confidence interval.

Data in bold indicate statistical significance; ^*^ *p* < 0.05, ^**^p < 0.01, ^***^ *p* < 0.001.

^a^ Linear regression analysis was utilized to assess the relationship between baseline HDL-C levels and the likelihood of cognitive impairment.

### 3.3. Nonlinear association

**Fig 2:**
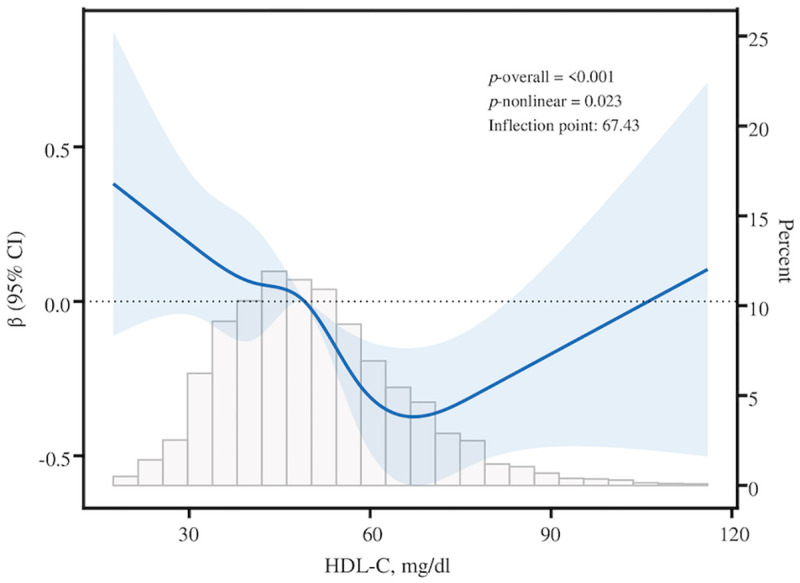
Restricted cubic spline for the association between HDL-C (mg/dL) and cognitive performance. Models adjusted as described in Statistical Analysis.

By employing RCS curves with covariate adjustment in Model III, all participants exhibited a nonlinear relationship between HDL-C level and dementia risk (*p* for nonlinearity = 0.023) (Fig 2). A subsequent investigation of the impact of the saturation threshold (Table 3) indicated that the inflection point was 67.43. Before the inflection point, a significant inverse link was noted, however a direct correlation was established beyond this threshold. Specifically, beneath the threshold, each unit increase in HDL-C was associated with an 8% reduction in the probability of cognitive deterioration (95% CI, −0.11 to −0.06; *p* < 0.001). Subsequent to the inflection point, each incremental unit of HDL-C was associated with a 7% increased risk of cognitive decline (95% CI, 0.01 to 0.14, *p* = 0.019). These continuous models characterize the exposure–response across the full HDL-C range and identify a turning point at 67.43 mg/dL, with inverse associations below and positive associations above this threshold. Apparent differences from Section 3.2 arise from reference-based categorization (Q1 as referent) versus continuous modeling (splines/threshold), which are complementary and together indicate lower risk at moderate HDL-C and higher risk at very high HDL-C.

**Table 3:** Regression Analysis of Threshold Effects: Impact of standardized HDL-C values on cognitive function assessments.

Characteristic	Beta per SD	95% CI	*p*-value
HDL-C (< 67.43)	−0.08	−0.11, −0.06	<0.001
HDL-C (≥ 67.43)	0.07	0.01, 0.14	0.019

CI = Confidence Interval, ^*^p<0.05, ^**^p<0.01, ^***^p<0.001.

Beta coefficients were adjusted for covariates such as age, gender, educational attainment, marital status, hypertension, diabetes, depressive symptoms, alcohol consumption, smoking behaviours, social engagement, total cholesterol, and triglyceride levels.

### 3.4. Subgroup analysis

**Fig 3:**
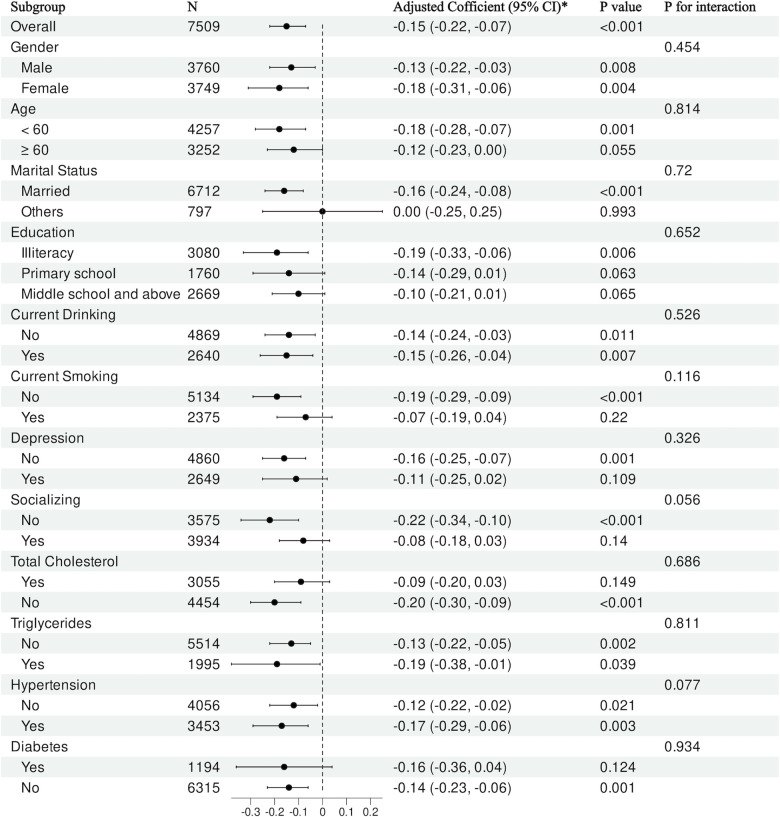
Subgroup analyses for the association between HDL-C and cognitive impairment. Models adjusted as described in Statistical Analysis; *p*-values for interaction are shown where applicable.

A substantial negative association (β = −0.15; 95% CI, −0.22 to −0.07; p < 0.001) indicates that higher HDL-C is associated with a lower probability of cognitive impairment in the overall model. After adjusting for sex, age, marital status, educational attainment, smoking habits, alcohol intake, depression, social engagement, total cholesterol, triglycerides, hypertension, and diabetes, the relationship between HDL-C levels and the risk of cognitive decline exhibited no statistically significant alterations (all p > 0.05). This suggests that the risk of cognitive impairment identified in the general population was uniform across various subgroups (Fig 3).

### 3.5. Sensitivity analysis using the non-HDL-C/HDL-C ratio (NHHR)

To consider HDL-C within the broader lipid profile, we further examined the non-HDL-C/HDL-C ratio (NHHR). In the fully adjusted model, each 1-SD increase in NHHR was associated with a higher cognitive score (β = 0.154; 95% CI, 0.059–0.250; p = 0.001). Compared with participants in the lowest NHHR quartile, those in Q2–Q4 had progressively higher cognitive scores (Q2: β = 0.196; 95% CI, 0.007–0.385; p = 0.042; Q3: β = 0.278; 95% CI, 0.085–0.472; p = 0.005; Q4: β = 0.372; 95% CI, 0.148–0.596; p = 0.001; p for trend < 0.001) (Table 4).

**Table 4:** Sensitivity analysis: association between NHHR (non-HDL-C/HDL-C ratio) and cognitive score.

Exposure	Model I	Model II	Model III
	β (95% CI), p-value	β (95% CI), p-value	β (95% CI), p-value
NHHR			
Per SD increase	**0.246 (0.166–0.326)** <0.001***	**0.235 (0.160–0.310)** <0.001***	**0.154 (0.059–0.250)** 0.001**
NHHR quartiles			
Q1	Reference	Reference	Reference
Q2	**0.330 (0.104–0.555)** 0.004**	**0.364 (0.151–0.576)** <0.001***	**0.196 (0.007–0.385)** 0.042*
Q3	**0.468 (0.243–0.694)** <0.001***	**0.484 (0.271–0.697)** <0.001***	**0.278 (0.085–0.472)** 0.005**
Q4	**0.722 (0.496–0.947)** <0.001***	**0.727 (0.514–0.939)** <0.001***	**0.372 (0.148–0.596)** 0.001**
*p* for trend	<0.001***	<0.001***	<0.001***

Model I: unadjusted; Model II: adjusted for age and sex; Model III: further adjusted for education, marital status, hypertension, diabetes, depressive symptoms, alcohol consumption, smoking behaviours, social engagement, and triglyceride levels. NHHR = (total cholesterol – HDL-C)/HDL-C. CI: Confidence interval. Data in bold indicate statistical significance; *p < 0.05, **p < 0.01, *** *p* < 0.001.

## 4. Discussion

A cross-sectional study involving 7,509 middle-aged and elderly Chinese individuals indicated that elevated HDL-C levels (60–70 mg/dL and over 70 mg/dL) correlated with an increased probability of cognitive dysfunction. This result challenges the conventional belief that elevated HDL-C levels serve as protective factors against cardiovascular and cerebrovascular diseases. Our findings provide new evidence that elevated HDL-C levels may adversely affect cognitive impairment in middle-aged and older adults. Consistent with the threshold analysis (inflection at 67.43 mg/dL), below the threshold each unit increase in HDL-C was associated with an 8% lower probability of cognitive impairment, whereas above the threshold each unit increase was associated with a 7% higher probability of impairment.. Beyond static HDL-C concentrations, HDL functionality is increasingly recognized as more biologically relevant. Key properties include cholesterol efflux capacity (via ABCA1/ABCG1 and SR-BI) and anti-inflammatory/anti-oxidative as well as endothelial effects; these may not rise linearly with HDL-C and can even diverge from concentration at higher ranges [15,17]. Age-related inflammation and oxidative stress can remodel HDL into a dysfunctional phenotype with impaired efflux and anti-oxidative capacity, providing a plausible explanation for the upward limb of our U-shaped association at very high HDL-C.

The relationship between HDL-C concentrations and cognitive dysfunction remains a topic of ongoing discussion. Previous studies have indicated that HDL-C has a protective effect on brain health and neurodegenerative disorders. Elevated concentrations of small-particle HDL-C and enhanced HDL efflux capacity may mitigate neurotoxicity associated with excess cholesterol in the central nervous system [30,31]. A significant positive linear correlation between HDL-C concentrations and MMSE scores was identified in previous research conducted in China through linear regression analysis. The model exhibited a substantial reduction in the risk of cognitive dysfunction with increased HDL-C levels (odds ratio [OR], 0.81; 95% CI, 0.70 to 0.94) following a complete adjustment [32]. Additionally, a cross-sectional study of HDL-C subgroups indicated a positive correlation between HDL3-C and global comprehensive cognitive tests (*p* = 0.03), whereas HDL2-C showed no correlation with memory tests (*p* > 0.05) [33]. Recently, another report by Hussain, S. M et al. indicated that elevated HDL-C levels (particularly >80 mg/dL) positively correlated with all-cause dementia (hazard ratio [HR], 1.27; 95% CI, 1.03 to 1.58), with a more pronounced association observed in women (HR, 1.42; 95% CI, 1.10 to 1.83) [34]. Similarly, Ferguson et al. reported that both elevated (>65 mg/dL) and reduced (11–41 mg/dL) HDL-C concentrations were associated with a higher risk of cognitive dysfunction, with risk ratios of 1.15 (95% CI, 1.11 to 1.20) and 1.07 (95% CI, 1.03 to 1.11), respectively [35]. Findings from a multicenter, randomized, double-blind, placebo-controlled phase 3 clinical trial indicated that increasing HDL-C levels had a minimal effect on lowering the risk of cardiovascular disease (12.9%), similar to the placebo group (12.8%) [36]. Another pharmacological study yielded comparable findings [37]. This may explain why elevated HDL-C levels are not regarded as beneficial to cholesterol, contrary to traditional beliefs. Therefore, HDL-C provides limited protection to cognitive function.

Our study identified a U-shaped nonlinear association between HDL-C levels and cognitive impairment. HDL-C, an essential component of serum lipoproteins, is frequently referred to as “good cholesterol” because of its role in cholesterol metabolism and its anti-inflammatory and anti-thrombotic properties [38]. Reduced HDL-C levels are correlated with a heightened risk of cognitive impairment. A Danish cohort study revealed that diminished HDL-C levels were significantly linked to adverse outcomes in persons with chronic renal illness, cancer, or inflammatory disorders, irrespective of cardiovascular disease [39]. A Mendelian randomization study on blood lipids and cognitive impairment indicated that low HDL-C levels correlated with an increased risk of dementia (OR, 1.20; 95% CI, 1.03 to 1.40). Notably, these results are consistent with our findings. In sensitivity analyses using NHHR, higher NHHR was associated with higher cognitive scores, suggesting that a higher HDL-C fraction (i.e., lower NHHR) is not uniformly beneficial across the full lipid spectrum. This finding is consistent with our observed nonlinear (U-shaped) association, particularly the potential adverse association at very high HDL-C levels (Table 4).

Apparent inconsistencies in the HDL-C–cognition literature can be explained by several design and analytic dimensions. Population factors (age/sex composition, APOE ε4, vascular and inflammatory burden, medications) influence both HDL biology and cognitive risk [16,34]. Outcome definitions vary (global vs. domain-specific cognition; continuous scores vs. clinical impairment), affecting sensitivity to nonlinearity and floor/ceiling effects [32,35]. Exposure handling differs across studies (single measurement vs. longitudinal trajectories; fasting vs. non-fasting sampling; categorical cut-points and referent choice vs. restricted cubic splines/thresholds) [22,34]. Modeling choices (confounder sets, potential over-/under-adjustment, and a-priori specification of nonlinearity) further shape direction and magnitude. Finally, biology matters: HDL-C concentration alone may not capture HDL functionality (e.g., efflux, anti-inflammatory/anti-oxidative actions), and inflammatory milieus may foster dysfunctional HDL at very high HDL-C [10,15,40]. Considering these dimensions reconciles seemingly divergent reports while supporting a nonlinear association with potential harms at very high HDL-C and benefits in moderate ranges.

Nonetheless, the correlation between elevated HDL-C levels and cognitive decline, along with the underlying physiological mechanisms, remains poorly understood. Yetukuri et al. discovered that individuals with both low and elevated HDL-C concentrations display different HDL particle compositions [41]. NMR-based lipidomic analysis indicated that, alongside variations in HDL lipid profiles, the high HDL-C group exhibited enrichment of sphingomyelin and free cholesterol on the surface [42]. Furthermore, elevated HDL-C levels may result in dysfunctional HDL particles and a transition from anti-inflammatory to pro-inflammatory effects [40,43]. Chronic brain inflammation can result in the buildup of detrimental proteins, which potentially facilitate the onset of various dementias, such as Alzheimer’s disease [44,45]. Moreover, genetic variants linked to elevated HDL-C levels may result in an increased occurrence of adverse cardiovascular and cerebrovascular events [42]. Further investigation is required into the potential pathological mechanisms underlying cognitive decline associated with elevated HDL-C levels. With respect to cognitive outcomes, HDL particle heterogeneity may be relevant: smaller, apoA-I–rich subfractions have been linked to more favorable vascular and neuroinflammatory profiles, whereas dysfunctional HDL may fail to confer these benefits at very high HDL-C. Such functionality-based mechanisms align with our findings and with reports connecting HDL features to cognitive phenotypes [30,31,33].

While our study offers significant insights, it has several limitations. First, because CHARLS is observational, we cannot establish a causal relationship between HDL-C and cognitive function. Second, the cognitive assessment may not comprehensively capture all cognitive domains. Third, despite multivariable multivariate adjustments, unmeasured confounding—such as dietary patterns and physical activity—may persist. In addition, owing to the cross-sectional design, we cannot determine the long-term effects of changes in HDL-C on cognitive performance; longitudinal studies are warranted to evaluate whether trajectories of HDL-C over time predict cognitive decline. Finally, we did not measure HDL functionality (e.g., cholesterol efflux capacity, paraoxonase-1 activity, anti-oxidative/anti-inflammatory indices) or HDL subfractions, precluding a direct test of whether dysfunctional HDL at very high HDL-C underlies the observed U-shape.

## 5. Conclusion

Our findings linked HDL-C levels and the chance of cognitive impairment in a U-shaped manner. These results suggest that both low and high HDL-C levels may damage the brains of older people. In particular, it is crucial that older adults with high HDL-C levels regularly assess their cognitive abilities. Further studies are needed to determine the possible pathophysiological processes underlying the effects of different HDL-C particles or subfractions on brain function.
